# 549. Ensitrelvir for the Treatment of COVID-19 Infection: Evaluation of Taste Disorder and Smell Disorder in the Phase 3 Part of the Phase 2/3 SCORPIO-SR Randomized Controlled Trial

**DOI:** 10.1093/ofid/ofad500.618

**Published:** 2023-11-27

**Authors:** Yuko Tsuge, Takuhiro Sonoyama, Hiroshi Yotsuyanagi, Norio Ohmagari, Yohei Doi, Masaya Yamato, Takumi Imamura, Hiroshi Mukae

**Affiliations:** Shionogi, Osaka, Osaka, Japan; Shionogi & Co., Ltd., Osaka, Osaka, Japan; The University of Tokyo, Tokyo, Tokyo, Japan; National Centre for Global Health and Medicine, Shinjuku, Tokyo, Japan; Fujita Health University School of Medicine, Toyoake, Aichi, Japan; Rinku General Medical Center, Izumisano, Osaka, Japan; Shionogi, Osaka, Osaka, Japan; Nagasaki University, Nagasaki, Nagasaki, Japan

## Abstract

**Background:**

Ensitrelvir, a selective SARS-CoV-2 3CL protease inhibitor, is developed as an oral antiviral agent for the treatment of COVID-19 infection. In the Phase 3 part of the randomized SCORPIO-SR trial, the effects of oral ensitrelvir 125 mg and 250 mg on the resolution of taste and smell disorder in patients with COVID-19 with or without SARS-CoV-2 vaccination and risk factors for severe disease were evaluated.

**Methods:**

This study is a multicenter, randomized, double-blind, placebo-controlled study conducted in Japan, South Korea and Vietnam. SARS-CoV-2-positive patients, aged 12–70 years old, received ensitrelvir 125 mg PO (after 375 mg PO loading dose on Day 1 only), 250 mg PO (after 750 mg PO loading dose on Day 1 only) or placebo, once daily for 5 days, and were followed by Day 21 from start of treatment to analyze the proportion of patients presenting with taste or smell disorder.

**Results:**

55.6% of patients were male in the ensitrelvir 125 mg group (N=347), 54.4% in the 250 mg group (N=340), and 50.7% in the placebo group (N=343). The mean (standard deviation) age was 35.7 (12.5) years in the ensitrelvir 125 mg group, 35.3 (12.2) years in the 250 mg group, and 34.7 (12.2) years in the placebo group among patients for whom the time from onset to randomization was less than 72 hours. The proportions of patients with taste disorder or smell disorder were smaller in both the 125 mg and 250 mg groups compared with the placebo group on Day 5 to Day 9 (Figure1). Significantly smaller proportions of patients had taste disorder or smell disorder on Day 7 and Day 8 in the 125 mg ensitrelvir group, and on Day 8 and Day 9 in the 250 mg ensitrelvir group compared with the placebo group, respectively. In the group of patients who did not have taste disorder or smell disorder at the start of treatment, the proportions of patients who developed taste or smell disorder were smaller in both the 125 mg and the 250 mg groups compared with the placebo group after 4 days from the start of treatment (Figure2).

**Figure:**
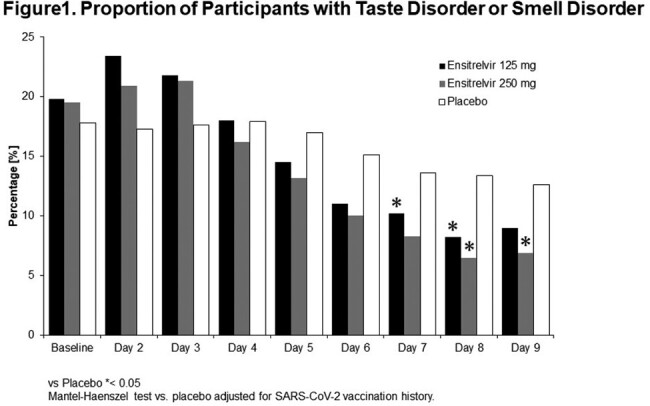

**Figure:**
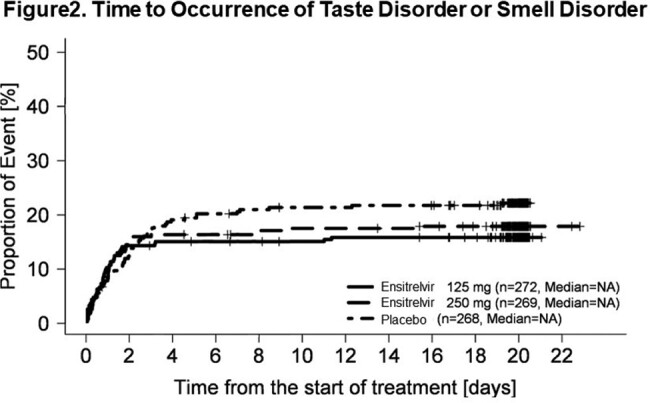

**Conclusion:**

Early administration of oral ensitrelvir was associated with early improvement or prevention of taste disorder and smell disorder.

**Disclosures:**

**Yuko Tsuge, MSc**, SHIONOGI & CO., LTD.: Stocks/Bonds **Takuhiro Sonoyama, MD**, SHIONOGI & CO., LTD.: I am an employee of SHIONOGI & CO., LTD.|SHIONOGI & CO., LTD.: Stocks/Bonds **Hiroshi Yotsuyanagi, MD PhD**, Shionogi: Advisor/Consultant|Shionogi: Advisor/Consultant **Yohei Doi, MD, PhD**, bioMerieux: Advisor/Consultant|FujiFilm: Advisor/Consultant|Gilead: Advisor/Consultant|Gilead: Honoraria|GSK: Advisor/Consultant|Meiji Seika Pharma: Advisor/Consultant|Moderna: Advisor/Consultant|Moderna: Honoraria|MSD: Advisor/Consultant|MSD: Honoraria|Shionogi: Advisor/Consultant|Shionogi: Grant/Research Support|Shionogi: Honoraria **Masaya Yamato, MD, PhD**, Shionogi: Advisor/Consultant

